# C1GALT1 expression is associated with galactosylation of IgA1 in peripheral B lymphocyte in immunoglobulin a nephropathy

**DOI:** 10.1186/s12882-019-1675-5

**Published:** 2020-01-15

**Authors:** Yue Xing, Lina Li, Yaru Zhang, Fanghao Wang, Dandan He, Youxia Liu, Junya Jia, Tiekun Yan, Shan Lin

**Affiliations:** Department of Nephrology, General Hospital of Tianjin Medical University, Anshan road, Heping district, Tianjin, 154 NO China

**Keywords:** IgA nephropathy, Galactose-deficient IgA1, C1GALT1, β1, 3-galactosyltransferase, Meta-analysis

## Abstract

**Background:**

More and more studies demonstrated that genetic variation at C1GALT1 influences Gd-IgA1 level in IgAN. However, whether the expression of β1, 3-galactosyltransferase (β1, 3Gal-T) was influenced may provide insights into how Gd-IgA1 levels are controlled in IgAN.

**Methods:**

Thirty IgAN patients diagnosed in Tianjin Medical University General Hospital from April to September 2018 and 30 healthy volunteers whose age and gender matched with patients were enrolled in this study. Total Gd-IgA1 levels in plasma were determined by ELISA and C1GALT1 levels were determined by RT-PCR. Four databases (PubMed, EMBASE, CNKI, WanFang Medical Network) were searched to identify eligible studies that evaluated a difference in the expression of C1GALT1 in IgAN patients compared with total controls (non-IgAN and health controls). The C1GALT1C1 expression levels, which was indispensable to β1, 3Gal-T of IgA1, was also been compared.

**Results:**

Gd-IgA1 levels were remarkable higher in IgAN patients compared with healthy control. The expression levels of C1GALT1 gene were remarkably down-regulated in IgAN patients compared with healthy control. And the mRNA level of C1GALT1 was inversely correlated to Gd-IgA1 levels. In meta-analysis, six articles including 316 participants that analyzed the expression of β1, 3Gal-T were met inclusion criteria. There was no significant difference in the expression of C1GALT1 between IgAN patients compared with controls. And we found patients with IgAN had lower levels of C1GALT1 gene expression in the B cells compared to controls. The C1GALT1C1 levels in the IgAN patients were not different from the levels in the control group, which were unchanged no matter according to different ethnic population, different control group and different cell source. Two studies including 46 persons compared enzymatic activity of β1, 3Gal-T in B cells, and the result showed the β1, 3Gal-T activity was decreased in B cells.

**Conclusions:**

We found expression levels of C1GALT1 were remarkably downregulated in IgAN patients and negatively correlated with higher levels of Gd-IgA1. Subsequent meta-analysis validated the low expression and activity of β1, 3Gal-T in B cells in patients with IgAN. However, there was no apparent disparity in the aspect of C1GALT1C1 expression between IgAN and control groups.

## Background

IgA nephropathy (IgAN) is a common primary glomerular nephritis, which is considered to be a progressive disease characterized by a gradually decreasing glomerular filtration rate (GFR). The end-stage renal disease (ESRD) events happened in 15 to 20% of patients within 10 years [1, 2]. In the circulation of most patients with IgAN, galactose-deficient IgA1 (Gd-IgA1) and its corresponding autoantibodies are elevated and correlated with increased risk of disease progression. More and more studies demonstrated that genetic variation at C1GALT1 influences Gd-IgA1 level in IgAN [3]. C1GALT1 encodes β1, 3 galactosyltransferase (β1, 3Gal-T) enzyme which is important in O-galactosylation of glycoproteins. Whether β1, 3Gal-T was influenced may provide insights into how Gd-IgA1 levels are controlled. Some studies confirmed C1GALT1 gene expression level was significantly lower in IgAN patients than that of the control [4], however, the results of Qin W et al. did not support this view [5]. Enzyme activity was also compared between IgAN and control in several studies. These studies have yielded inconsistent results. Cosmc, encoded by C1GALT1C1, is core β1, 3Gal-T specific chaperone, and important for IgA1 glycosylation. Cosmc expression was previously reported to be down-regulated and associated with IgA1 glycosylation. Therefore, we comparatively assessed whether C1GALT1 down-regulated expression induced IgA1 aberrant glycosylation in IgAN and performed a meta-analysis to explore the expression and activity of β1, 3Gal-T in IgAN.

## Methods

### Subjects and data collection

Thirty IgAN patients diagnosed in Tianjin Medical University General Hospital from April to September 2018, were enrolled in this study. The diagnosis was based on the deposition of IgA in the glomerular mesangium by immunofluorescence detection, as well as the lack of clinical or serological evidence of other inflammatory conditions, such as Henoch Schoenlein purpura, systemic lupus erythematosus. At the same time, 30 healthy volunteers whose age and gender matched with patients were recruited. The estimated glomerular filtration rate (eGFR) was calculated using the Chronic Kidney Disease Epidemiology Collaboration creatinine (CKD-EPI) equation. The histological lesions were classified according to Oxford classification system.

Plasma was collected from all individuals in this study, for patients at the time of renal biopsy. The plasma samples were stored in aliquots at − 80 °C for the subsequent use. Clinical information, including age, gender, 24-h urine protein excretion, blood pressure and total IgA levels, were collected at the time of renal biopsy.

### Assay for Gd-IgA1

Plasma Gd-IgA1 levels were detected using ELISA kit according to the manufacturer’s specifications (IBL, Japan) [6]. Plasma samples were diluted in proportions of 1:50 with EIA buffer and incubation for 60 min at R.T. with plate lid. Then washing four times with wash buffer prepared labeled antibody were incubated for 30 min. Plates were washed and added 50ul TMA solution incubation for 30 min in dark. At last, the color reaction was stopped and the absorbance was measured at 450/625 nm with an EL312 Bio-Kinetics microplate reader (Bio-TekInstruments, Winooski, VT).

### B lymphocytes isolation

About 5 mL venous blood sample was taken into ethylenediaminetetraacetic acid (EDTA) anticoagulated tubes. Peripheral blood mononuclear cells (PBMCs) were separated by density-gradient centrifugation on Ficoll (TBD, China), then washed three times with phosphatebuffered saline (PBS) and resuspended in PBS + 1% bovine serum albumin (BSA). Peripheral B lymphocytes were isolated using CD19 positive magnetic beads (Miltenyi Biotec, USA) according to the manufacturer’s instructions.

### RNA extraction

Total cellular RNA was extracted from CD19 positive B lymphocytes using the TRIZOL Reagent (Invitrogen, USA). RNA quantity was determined using NanoDrop ND-1000 spectrophotometer. cDNA was synthesized using 300 ng total RNA with revert first-strand cDNA kit according to manufacturer’s protocol (Promega, USA).

### Quantitative reverse transcription PCR (RT-PCR)

cDNA was amplified with a 20 μL reaction mixture using SYBR Green PCR Master Mix (Roche, USA) in an Applied Biosystem 7500 Real-Time PCR System. GAPDH served as the endogenous control. The primer sequences were listed in Table 1. The fold change between patients and controls was expressed by the 2^-△△CT^ method.

**Table 1:** Primers Used to Amplify the C1GALT1 and GAPDH Genes

Gene	Forward Primers (5′-3′)	Reverse Primers (5′-3′)
C1GALT1	TCCTCTGTGGATCAGCAATAGG	TTAGGCTGGGTGTCAACCTTT
GAPDH	TTGCCCTCAACGACCACTTT	TGGTCCAGGGGTCTTACTCC

### Ethics statement

The Medical Ethics Committee of Tianjin medical university general hospital approved the study protocol and informed written consent was obtained from all individuals. All study participants provided written informed consent for collection of blood samples, and for examination of clinical records relevant to the study.

### Meta-analysis

Eligible studies were included if all criteria were met as follows: (1) studies aimed at patients with IgAN, which were diagnosed with primary IgAN based on immunofluorescent and light microscopic; (2) the primary/original purpose of the study was to compare at least one of the following outcomes: the C1GALT1, C1GALT1C1 mRNA levels or β1, 3Gal-T activity; (3) all studies conducted in IgAN patients and controls. Four databases (PubMed, EMBASE, CNKI, Wanfang Medical Network) were searched from their earliest records to August 15th, 2018. No restriction on language or publication status was applied. The details of search keywords included “IgA glomerulonephritis”, “IgA nephropathy” “Glomerulonephritides”, “Berger’s disease”, “core-1β1–3 galactosyltransferase”, “C1GALT1 mRNA” “C1GALT1 expression”, “C1GALT1C1 mRNA” “C1GALT1C1 expression”, “β-galactosyltransferase expression “β-galactosyltransferase activity” and “controlled clinical trial”. Article without original data should be excluded. The main reasons for exclusion of trials are described in Additional file 1: Figure S1.

Two reviewers independently extracted the data from each study, collected data separately and finally summarized. The following related variables of patients were collected: country, mean age, male, sample size and detection index) (Table 2).

**Table 2:** Characteristics of the studies included in meta-analysis

Country	Mean age	Male(%)	Sample size	detection index
China [8]	NA	35 (57.4%); NA	IgAN 61; Healthy Control 65	C1GALT1 expression
China [9]	14.68; 9.04; 13.27	12 (52.1%); 5 (20%); 17 (73.9%)	IgAN 23; Control 33(Non- IgAN 10; Healthy Control 23)	C1GALT1 expression
UK [10]	44.5; 37	10 (83.3%); 7 (53.8%)	IgAN 12; Non- IgAN 13	C1GALT1 expression and β1, 3Gal-T activity
UK [11]	51.2; 43	3 (60%); 2 (40%)	IgAN 5; Healthy Control 5	C1GALT1 expression
UK [12]	33; 38	6 (66.6%); 6 (50%)	IgAN 9; Healthy Control 12	β1, 3Gal-T activity
China [5]	27.53; 29.4; 25	14 (34.1%); 7 (33.3%); 9 (56.2%)	IgAN 41; Control 37 (Non- IgAN 21; Healthy Control 16)	C1GALT1 expression
China	38; 37	16 (53.3%); 15 (50%)	IgAN 30; Healthy Control 30	C1GALT1 expression and Gd-IgA1 levels

### Risk of Bias

The Newcastle-Ottawa Scale (NOS) was used to assess the quality of included studies by judging them using three board perspectives: the selection of study groups, the comparability of study groups, and the measurement of exposure in study groups [7].

### Statistical analysis

The differences in gene expression levels between groups were compared using ANOVA and *p* value < 0.05 was considered statistically significant. Statistical analysis was performed using SPSS 17.0 software. In the review, for the continuous measurement of C1GALT1, C1GALT1C1 expression and β1, 3Gal-T activity, we used the weighted mean difference and square deviation (SD) between groups. We analyzed heterogeneity beyond chance using the I^2^ statistic to describe the percentage of variability. We made graphic representations of potential publication bias using Begg’s Funnel plots of the natural logarithm of the RR versus its standard error (SE) and assessed them visually. A 2-sided *p* value less than 0.05 was considered statistically significant, and all statistical analyses were performed using Review Manager 5.3 software.

## Results

### Baseline clinical characteristics of patients with IgAN

There were 16 males and 14 females with average age of 39.5 years. The median of proteinuria was 1.32 g/d and mean eGFR was 85.45 mL/min/1.73 m^2^ of IgAN patients on biopsy. And the grading of the pathological lesions by Oxford classification is shown in Table 3.

**Table 3:** The Baseline Data for Patients With IgAN and Healthy Controls

Characters	Mean ± SD or n (%)		
	IgAN	Healthy Controls	p
Male/female	16/14	15/15	0.80
Age (mean ± SD, year)	38 ± 11	37 ± 14	0.84
SBP (mmHg)	125 ± 18	126 ± 16	0.67
Proteinuria (g/d, median, IQR)	1.32 (0.38–3.34)		
Total IgA (ug/mL, median, IQR)	2350 (2060–3472)		
eGFR (mL/min 1.73 m2)	85.45 ± 25.71		
Oxford classification			
M score (M0/M1)	6 (20) /24 (80)		
E score (E0/E1)	18 (60) /12 (40)		
S score (S0/S1)	16 (53.3) /14 (46.7)		
T score (T0/T1/T2)	12 (40) /10 (33.3) /8 (26.7)		
C score (C0/C1/C2)	9 (30) /14 (46.7) /7 (23.3)		

### Patients with IgAN had low expression level of C1GALT1

**Fig. 1:**
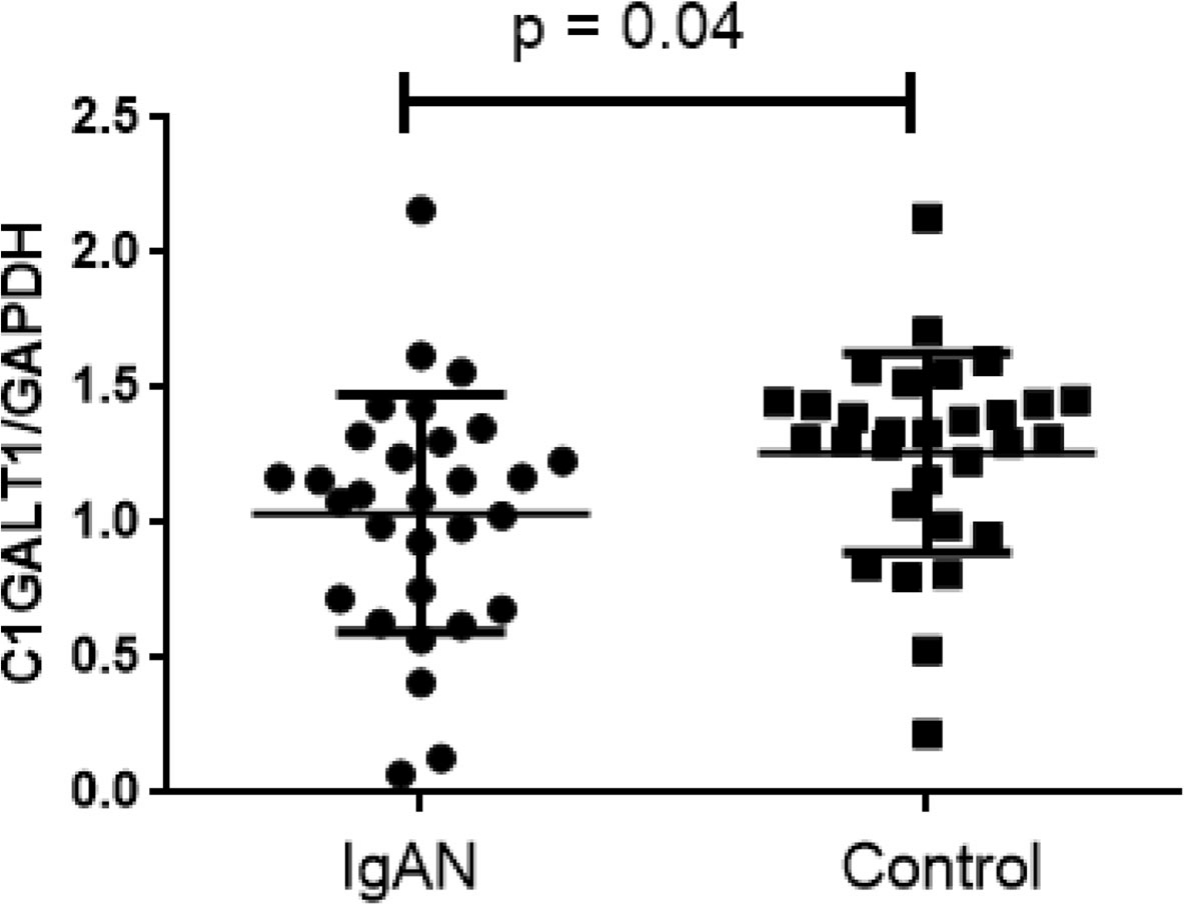
Expression level of C1GALT1 gene in IgAN and Control

The expression levels of C1GALT1 in B cells were detected in IgAN patients and healthy control. We found C1GALT1 expression levels were remarkably downregulated in IgAN patients (IgAN vs. controls: 1.01 ± 0.19 vs 1.43 ± 0.11, *p* = 0.04, Fig. 1).

### Expression of C1GALT1 related with the Gd-IgA1 levels

**Fig. 2:**
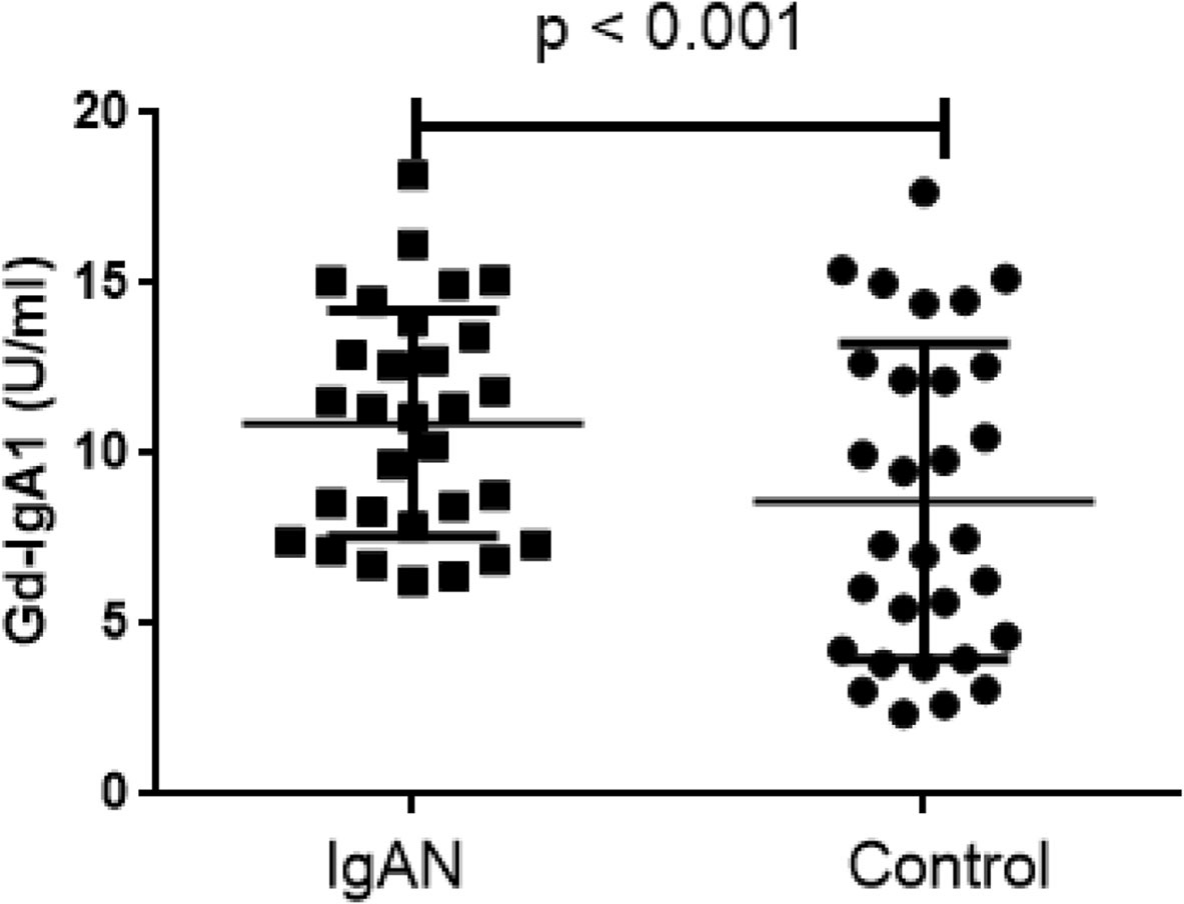
Gd-IgA1 levels in IgAN and Healthy Control

**Fig. 3:**
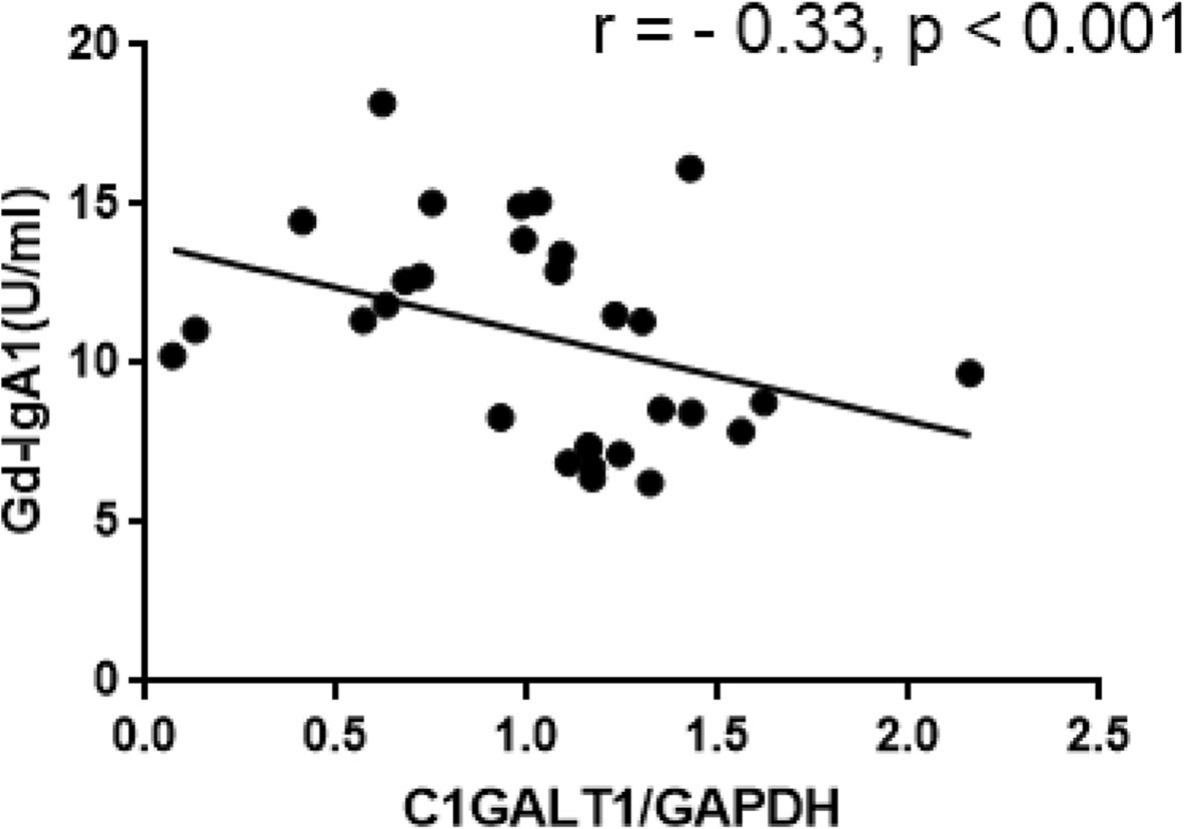
Expression of C1GALT1 related with the Gd-IgA1 levels

Using the GalNAc-specific monoclonal antibody KM55, we examined the plasma levels of Gd-IgA1 in patients with IgAN and healthy control. In our cohorts, the plasma level of Gd-IgA1 in patients with IgAN ranged from 8.55 to 14.48 U/mL, whereas it ranged from 3.97 to 12.15 U/mL in healthy control. We found that Gd-IgA1 levels were remarkable high in IgAN patients compared with healthy control (*p* < 0.001, Fig. 2). And the mRNA levels of C1GALT1 were inversely correlated to Gd-IgA1 levels (*r* = − 0.33, *p* < 0.001, Fig. 3).

### Meta-analysis

The literature search yielded 1052 articles, and eventually, six studies with 316 patients were included in our meta-analysis according to the inclusion criteria (Additional file 1: Figure S1). We included studies whose primary purpose was to elucidate the expression level and activity of C1GALT1 and the expression level of C1GALT1C1 in IgAN patients [5, 8–12]. Peripheral B lymphocyte was selected as target cell in 5 articles, and mononuclear cell in one article. Five studies compared the C1GALT1 and C1GALT1C1 mRNA expression levels and two measured the activity. Five studies were conducted in adult and one conducted in children. Of the contained six trials, four enrolled patients with IgAN and healthy control, two enrolled patients with IgAN, patients with non-IgAN glomerulonephritis and healthy control. The characteristics of the included studies were summarized in Table 2. These studies were performed from 1997 to 2016, with sample sizes ranging from 10 to 126.

### Quality assessment of included studies

The qualities of included studies, assessed with NOS (Newcastle-Ottawa Scale), are provided in Table 4. The mean total score was 5 with a range from 4 to 6.

**Table 4:** Quality assessment for the included trials

Reference	year	Selection of subjects/4*1	Comparability of groups/2*2	Measurement of Exposure/4*1	Total score of NOS/10
Shao [8]	2016	2	2	1	5
Cai [9]	2010	1	2	1	4
Buck [10]	2008	2	2	2	6
Boyd [11]	2014	2	2	1	5
Allen [12]	1997	1	2	1	4
Qin [5]	2005	2	2	2	6

Assessment of risk bias assessed with NOS (Newcastle-Ottawa Scale)

### C1GALT1 mRNA expression

**Fig. 4:**
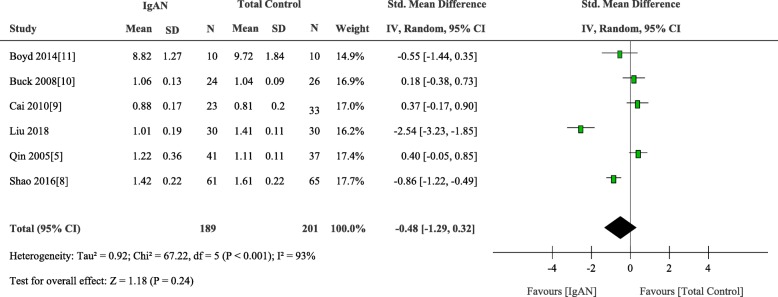
Comparison of the expression of C1GALT1 between IgAN and Total Control

**Fig. 5:**
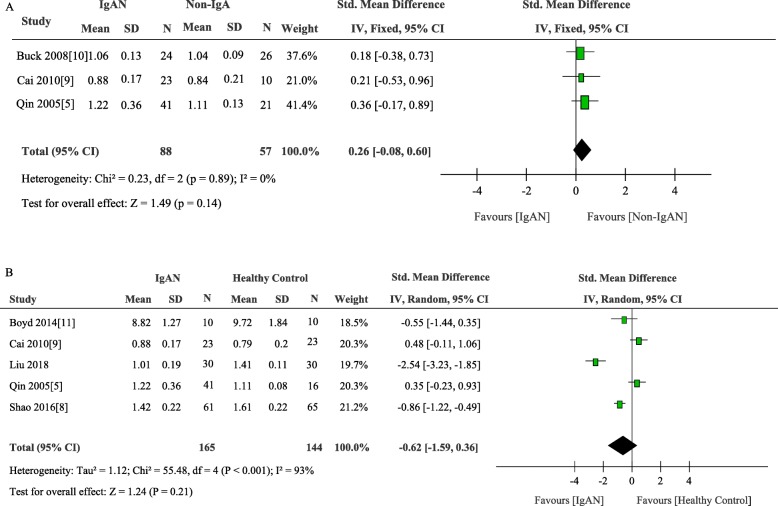
Comparison of the expression of C1GALT1 between IgAN and Non-IgAN (A) and Healthy Control(B)

We added own results to the final meta-analysis to elucidate the mRNA expression data of C1GALT1. Six articles including 316 participants that analyzed the expression of β1, 3Gal-T were included. There was no significant difference between IgAN patients and the total control group (weighted mean difference, − 0.48 [95% CI − 1.29 to 0.32], *p* = 0.24; I2 = 93%, p for heterogeneity < 0.001) (Fig. 4). Subgroup analysis was performed according to different control group and different cell source. Four studies compared mRNA expression level of C1GALT1 in 106 IgAN patients and 44 disease controls and five studies conducted in 160 IgAN patients and 139 healthy controls. There was no apparent disparity between IgAN patients and non-IgAN patients (weighted mean difference, 0.26 [95% CI − 0.08 to 0.60], *p* = 0.14; I2 = 0%, p for heterogeneity = 0.89) and healthy controls (weighted mean difference, − 0.62 [95% CI − 1.59 to 0.36], *p* = 0.21; I2 = 93%, p for heterogeneity < 0.001) (Fig. 5).

**Fig. 6:**
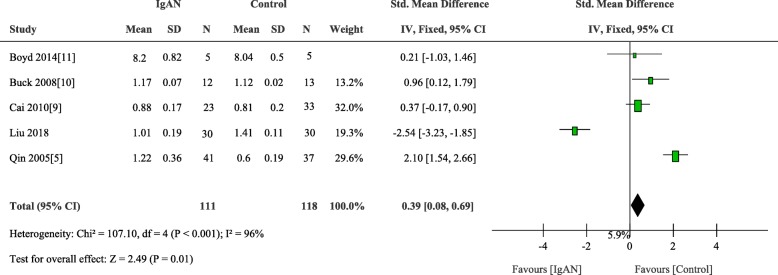
Comparison of the expression of C1GALT1 in B cells between IgAN and Total Control

In circulation, B lymphocytes are the major cells for IgA1 production. To explore the causal factor of the lymphocytes abnormality in patients with IgAN, in terms of β1, 3 galactosyltransferase induced Gd-IgA1 production, we detected the mRNA expression of C1GALT1 in B lymphocytes. A total of 4 studies enrolled 81 patients and 88 controls. Finding for this outcome was that patients with IgAN had low levels of C1GALT1 gene expression in the B lymphocytes compared to control (weighted mean difference, 0.39 [95% CI 0.08 to 0.69], *p* = 0.01; I2 = 96%, p for heterogeneity < 0.001) (Fig. 6).

### β1, 3Gal-T activity

**Fig. 7:**
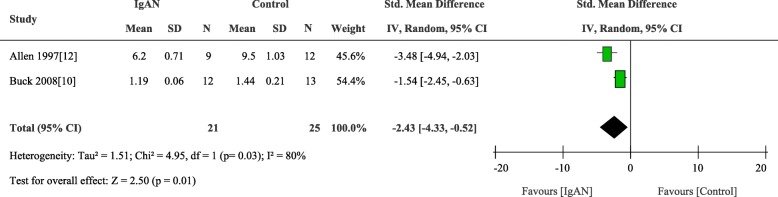
Comparison of the activity of β1, 3Gal-T in B cells between IgAN and Control

Comparison of β1, 3Gal-T activity was reported in 2 trials including 46 persons, the result showed patients with IgAN had low levels of β1, 3Gal-T activity in the B cells compared to control (weighted mean difference, − 2.43 [95% CI − 4.33 to − 0.52], *p* = 0.01; I^2^ = 80%, p for heterogeneity = 0.03) (Fig. 7).

### C1GALT1C1 expression

We also explored the expression of C1GALT1C1 in different groups. There was no significant difference between IgAN patients and the total control group (weighted mean difference, − 0.68 [95% CI − 1.61 to 0.25], *p* = 0.15; I2 = 93%, p for heterogeneity < 0.001) (Additional file 3: Figure S3). And the same result occurred between IgAN patients and non-IgAN patients (weighted mean difference, 0.20 [95% CI − 0.99 to 0.59], *p* = 0.62; I2 = 80%, p for heterogeneity = 0.007) and healthy control (weighted mean difference, − 0.80 [95% CI − 1.90 to 0.29], *p* = 0.39; I2 = 81%, p for heterogeneity = 0.002, Additional file 4: Figure S4). Even in the B lymphocytes the levels of C1GALT1C1 gene expression that between IgAN and the control group had no significant difference (weighted mean difference, − 0.34 [95% CI − 1.13 to 0.44], *p* = 0.39; I2 = 81%, p for heterogeneity = 0.002, Additional file 5: Figure S5). In the meantime, subgroup meta-analysis was conducted between Chinese and UK subjects. The result showed there were no different from the levels between different ethnic population (weighted mean difference, − 0.66 [95% CI − 2.09 to 0.77] vs − 0.65 [95% CI − 1.55 to 0.25], p for heterogeneity = 0.99, Additional file 6: Figure S6).

### Risk of bias

Formal statistical test showed that there was no publication bias in the expression of C1GALT1 compared between IgAN patients and total control (Begg’s test *p* = 0.452), which was showed in Additional file 2: Figure S2.

## Discussion

In recent years, the role of galactose-deficiency of IgA1 in IgAN is widely recognized, thus how to cause the occurrence of galactose-deficiency has become the focus of the research [13]. It demonstrates that common variation at C1GALT1 influences Gd-IgA1 level in IgAN [3, 14]. In this study we found expression levels of C1GALT1 were remarkably downregulated in IgAN patients and negatively correlated with higher levels of Gd-IgA1. Subsequent meta-analysis validated the low expression of C1GALT1 only in B cells in patients with IgAN.

The mechanisms leading to aberrant glycosylation of IgA1 in IgAN have been studied extensively; so far, two genome-wide association studies (GWAS) conducted in IgAN populations identified C1GALT1 that strongly associated with levels of Gd-IgA1, however, whether the mRNA level of C1GALT1 was associated with Gd-IgA1 levels in patients with IgAN needed to be determined. In the present study, we observed downregulated C1GALT1 mRNA’s expression in B lymphocytes in IgAN patients and patients with low C1GALT1 levels showed high levels of Gd-IgA1 in vivo. In circulation, CD19+ B lymphocytes are the major cells for IgA1 production. A study examined tonsillar B lymphocytes of IgAN showed the expression of C1GALT1 was significantly decreased in tonsillar CD19 positive B lymphocytes from IgAN patients compared to the control [15]. It might imply that B lymphocytes play an important role in deciding the C1GALT1 mRNA level, which suggested an abnormality of B lymphocytes in IgAN. Yamada et al. found IL-4 stimulation significantly decreased the mRNA levels of both C1GALT1 and Cosmc [5]. In Suzuki et al. showed IL-6 stimulation of IgA1-producing cells from IgAN patients accentuates galactose-deficiency of IgA1 O-glycans [16]. Investigation of the actual reason underlying the expression deficiency of C1GALT1 may shed light on the mechanism of IgAN and help to find new therapy of this disease. Many experts hold the reviews that down-regulation of C1GALT1 expression or activity of β1, 3Gal-T in B lymphocytes, which inducing increased production of aberrantly glycosylated IgA1 molecules, ultimately contributed to IgAN pathogenesis [17]. Thus, according to these studies and views, it seems to closely show that this downregulation might be one of the fundamental pathogenic abnormalities in IgAN.

In the meta-analysis, patients with IgAN had low β1, 3Gal-T activity in the B lymphocytes compared to control group. However, it is not clarified whether abnormalities of IgA1 O-glycosylation was a result from reduced β1, 3Gal-T activity, there is a paucity of information on the regulation of activity of this glycosyltransferase. Cosmc, encoded by C1GALT1C1, is core 1 beta-3-galactosyltransferase specific chaperone, and important for IgA1 glycosylation [18]. The expression of C1GALT1C1 was closely related to the activity of β1, 3Gal-T, and it was previously reported to be down-regulated and associated with IgA1 glycosylation [17]. Therefore we analyzed the expression of C1GALT1C1 by meta-analysis, the result showed that there was no significant difference between IgAN patients and the total control group. Furthermore, the results were not modified by different ethnic population, different control group and different cell source. Further studies were needed for a better understanding of the potential mechanism of the change of β1, 3Gal-T activity.

### Limitations

Our study does, however, have several limitations. Firstly, because of the lack of sufficient data, a subgroup analysis explored the heterogeneity between C1GALT1 and the pathogenesis of IgAN is not conducted. Secondly, the specific quantification methods of qPCR experiments and internal control differed between studies, which may affect the effect sizes. Thirdly, the sample size of our study is small and the involved studies have poor quality assessment. We will further expand the sample size to verify the expression of C1GALT1 in IgA patients and its relationship with Gd-IgA1 levels.

## Conclusion

We found expression level of C1GALT1 was remarkably downregulated in IgAN patients and negatively correlated with higher levels of Gd-IgA1. Subsequent meta-analysis validated the low C1GALT1 expression and activity of β1, 3Gal-T in B lymphocytes in patients with IgAN. Furthermore, the results showed there were no apparent disparities between IgAN and control groups in the aspect of C1GALT1C1 expression, even in peripheral B lymphocytes. Together, these results revealed an aberrant β1, 3Gal-T expression and activity in B cells from IgAN patients, which could be identified as potential targets for future disease specific therapy of IgAN.

## Supplementary information

**Additional file 1: Figure S1.** Process for identifying studies eligible for the meta-analysis. (PPTX 64 kb)

**Additional file 2: Figure S2.** Begg’s funnel plot of the expression of C1GALT1 between IgAN and Control. (PPTX 44 kb)

**Additional file 3: Figure S3.** Comparison of the expression of C1GALT1C1 between IgAN and Control. (PPTX 66 kb)

**Additional file 4: Figure S4.** Comparison of the expression of C1GALT1C1 between IgAN and Non-IgAN (A) and Healthy Control(B). (PPTX 88 kb)

**Additional file 5: Figure S5.** Comparison of the expression of C1GALT1C1 in B cells between IgAN and Total Control. (PPTX 64 kb)

**Additional file 6: Figure S6.** Comparison of the expression of C1GALT1C1 in different ethnic population between IgAN and Total Control. (PPTX 80 kb)

## Data Availability

Raw data used during the current study are available from the corresponding author on reasonable request for non-commercial use.

## Abbreviations

eGFR: Estimated glomerular fltration rate
ESRD: End-stage renal disease
Gd-IgA1: Galactose-defcient IgA1
GWAS: Genome-wide association study
IgAN: IgA nephropathy
NOS: (Newcastle-Ottawa Scale)
PBMC: Peripheral blood mononuclear cell
SNPs: Single nucleotide polymorphisms
β1, 3Gal-T: β1, 3-galactosyltransferase
