# Supplementary material for: C1GALT1 expression is associated with galactosylation of IgA1 in peripheral B lymphocyte in immunoglobulin a nephropathy
Source: BMC Nephrol. 2020 Jan 15;21:18. doi: 10.1186/s12882-019-1675-5 (PMC6964072; doi:10.1186/s12882-019-1675-5)
Supplement: Supplementary file 1 — Additional file 1: Figure S1. Process for identifying studies eligible for the meta-analysis. (PPTX 64 kb) [file 12882_2019_1675_MOESM1_ESM.pptx]

## Slide 1
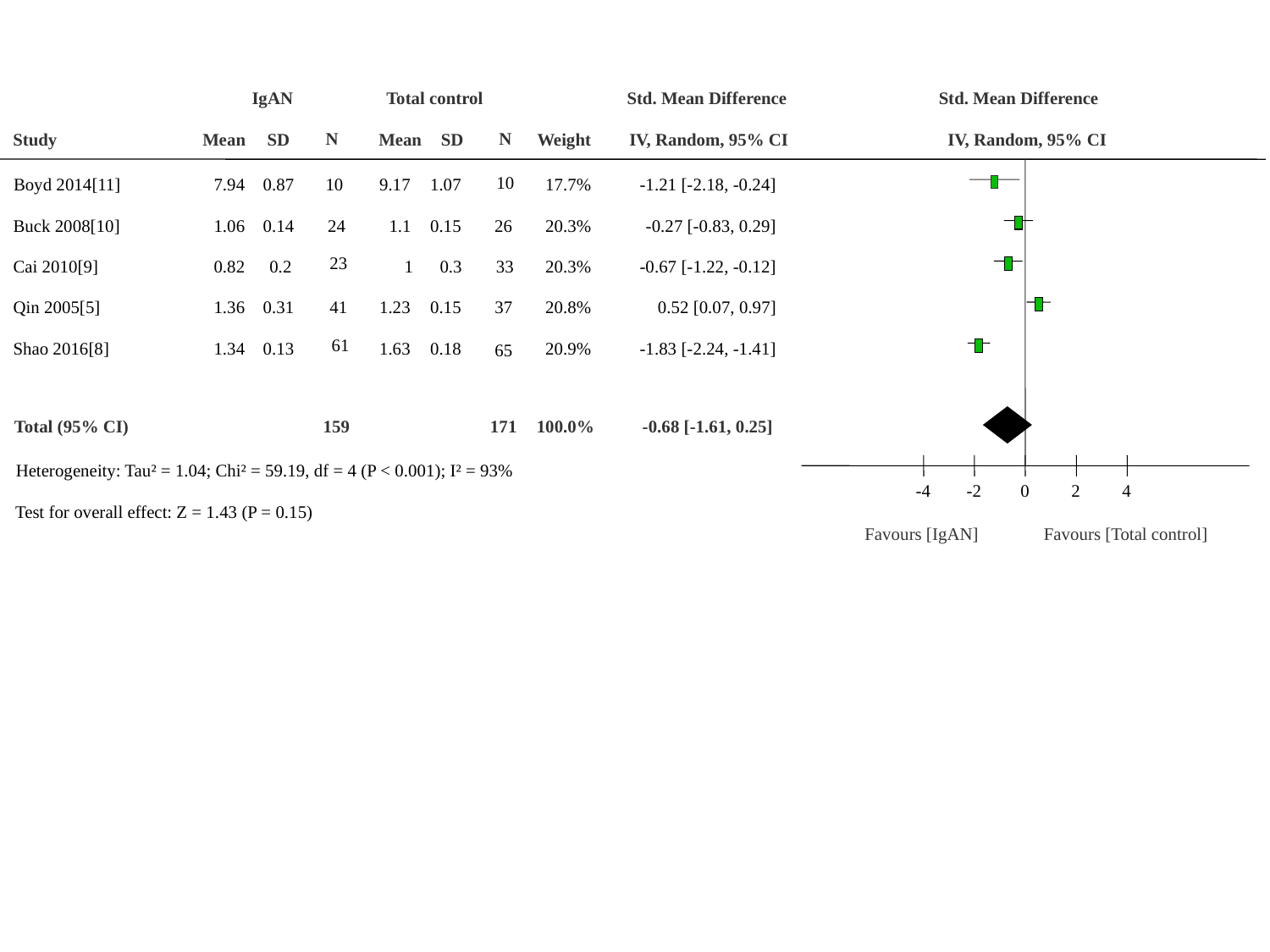

IgAN
Total control
Std. Mean Difference
Std. Mean Difference
N
N
Study
Mean
SD
Mean
SD
Weight
IV, Random, 95% CI
IV, Random, 95% CI
10
Boyd 2014[11]
7.94
0.87
10
9.17
1.07
17.7%
-1.21 [-2.18, -0.24]
Buck 2008[10]
1.06
0.14
24
1.1
0.15
26
20.3%
-0.27 [-0.83, 0.29]
23
Cai 2010[9]
0.82
0.2
1
0.3
33
20.3%
-0.67 [-1.22, -0.12]
Qin 2005[5]
1.36
0.31
41
1.23
0.15
37
20.8%
0.52 [0.07, 0.97]
61
Shao 2016[8]
1.34
0.13
1.63
0.18
20.9%
-1.83 [-2.24, -1.41]
65
Total (95% CI)
159
171
100.0%
-0.68 [-1.61, 0.25]
Heterogeneity: Tau² = 1.04; Chi² = 59.19, df = 4 (P < 0.001); I² = 93%
-4
-2
0
2
4
Test for overall effect: Z = 1.43 (P = 0.15)
Favours [IgAN]
Favours [Total control]
