# Supplementary material for: C1GALT1 expression is associated with galactosylation of IgA1 in peripheral B lymphocyte in immunoglobulin a nephropathy
Source: BMC Nephrol. 2020 Jan 15;21:18. doi: 10.1186/s12882-019-1675-5 (PMC6964072; doi:10.1186/s12882-019-1675-5)
Supplement: Supplementary file 2 — Additional file 2: Figure S2. Begg’s funnel plot of the expression of C1GALT1 between IgAN and Control. (PPTX 44 kb) [file 12882_2019_1675_MOESM2_ESM.pptx]

## Slide 1
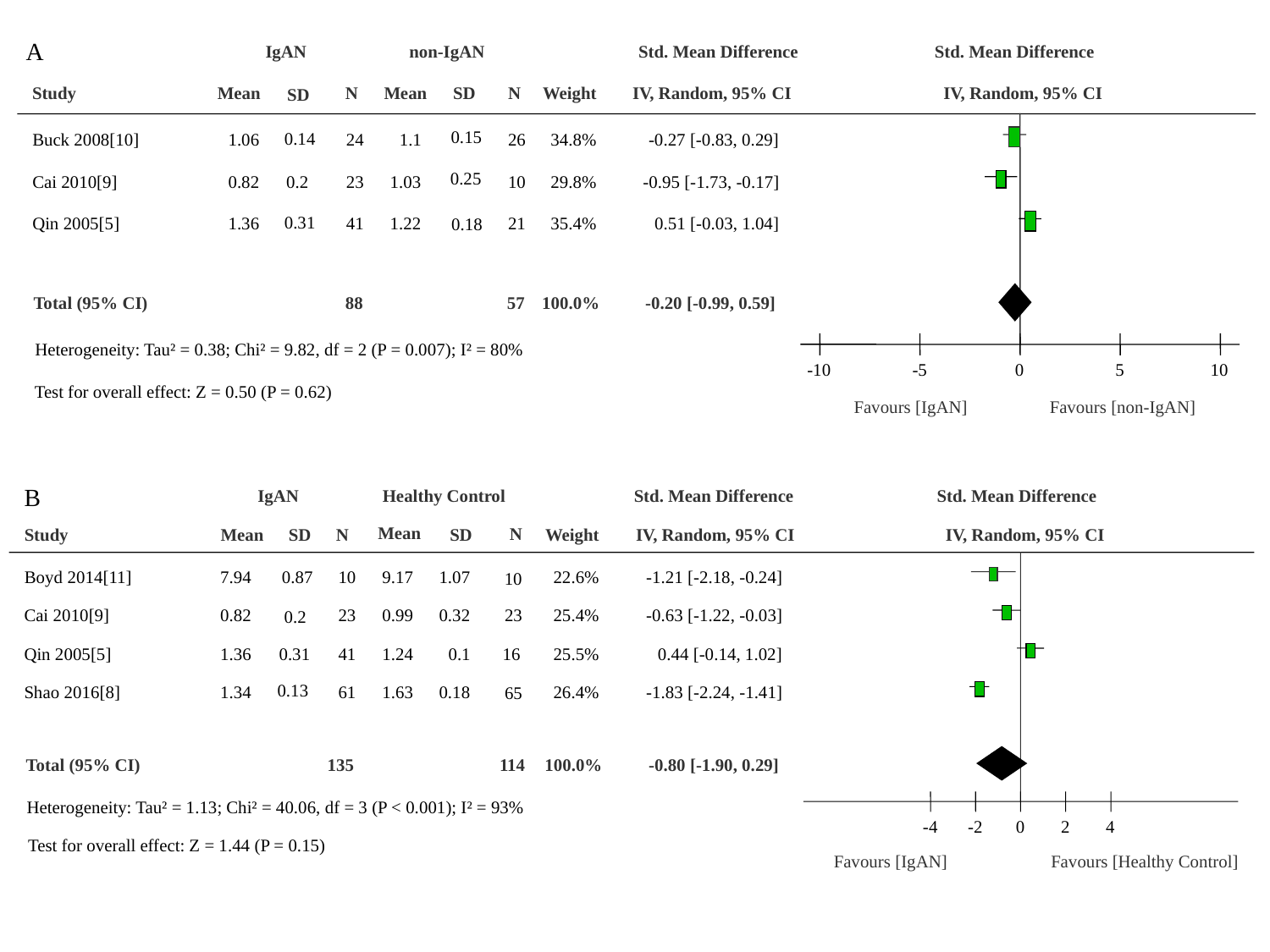

A
IgAN
non-IgAN
Std. Mean Difference
Std. Mean Difference
Study
Mean
N
Mean
SD
N
Weight
IV, Random, 95% CI
IV, Random, 95% CI
SD
0.15
0.14
Buck 2008[10]
1.06
24
1.1
26
34.8%
-0.27 [-0.83, 0.29]
0.25
Cai 2010[9]
0.82
0.2
23
1.03
10
29.8%
-0.95 [-1.73, -0.17]
0.31
Qin 2005[5]
1.36
41
1.22
21
35.4%
0.51 [-0.03, 1.04]
0.18
Total (95% CI)
88
57
100.0%
-0.20 [-0.99, 0.59]
Heterogeneity: Tau² = 0.38; Chi² = 9.82, df = 2 (P = 0.007); I² = 80%
-10
-5
0
5
10
Test for overall effect: Z = 0.50 (P = 0.62)
Favours [IgAN]
Favours [non-IgAN]
B
IgAN
Healthy Control
Std. Mean Difference
Std. Mean Difference
Mean
N
Study
Mean
SD
N
SD
Weight
IV, Random, 95% CI
IV, Random, 95% CI
Boyd 2014[11]
7.94
0.87
10
9.17
1.07
22.6%
-1.21 [-2.18, -0.24]
10
Cai 2010[9]
0.82
23
0.99
0.32
23
25.4%
-0.63 [-1.22, -0.03]
0.2
Qin 2005[5]
1.36
0.31
41
1.24
0.1
16
25.5%
0.44 [-0.14, 1.02]
0.13
Shao 2016[8]
1.34
61
1.63
0.18
26.4%
-1.83 [-2.24, -1.41]
65
Total (95% CI)
135
114
100.0%
-0.80 [-1.90, 0.29]
Heterogeneity: Tau² = 1.13; Chi² = 40.06, df = 3 (P < 0.001); I² = 93%
-4
-2
0
2
4
Test for overall effect: Z = 1.44 (P = 0.15)
Favours [IgAN]
Favours [Healthy Control]
