# Supplementary material for: C1GALT1 expression is associated with galactosylation of IgA1 in peripheral B lymphocyte in immunoglobulin a nephropathy
Source: BMC Nephrol. 2020 Jan 15;21:18. doi: 10.1186/s12882-019-1675-5 (PMC6964072; doi:10.1186/s12882-019-1675-5)
Supplement: Supplementary file 3 — Additional file 3: Figure S3. Comparison of the expression of C1GALT1C1 between IgAN and Control. (PPTX 66 kb) [file 12882_2019_1675_MOESM3_ESM.pptx]

## Slide 1
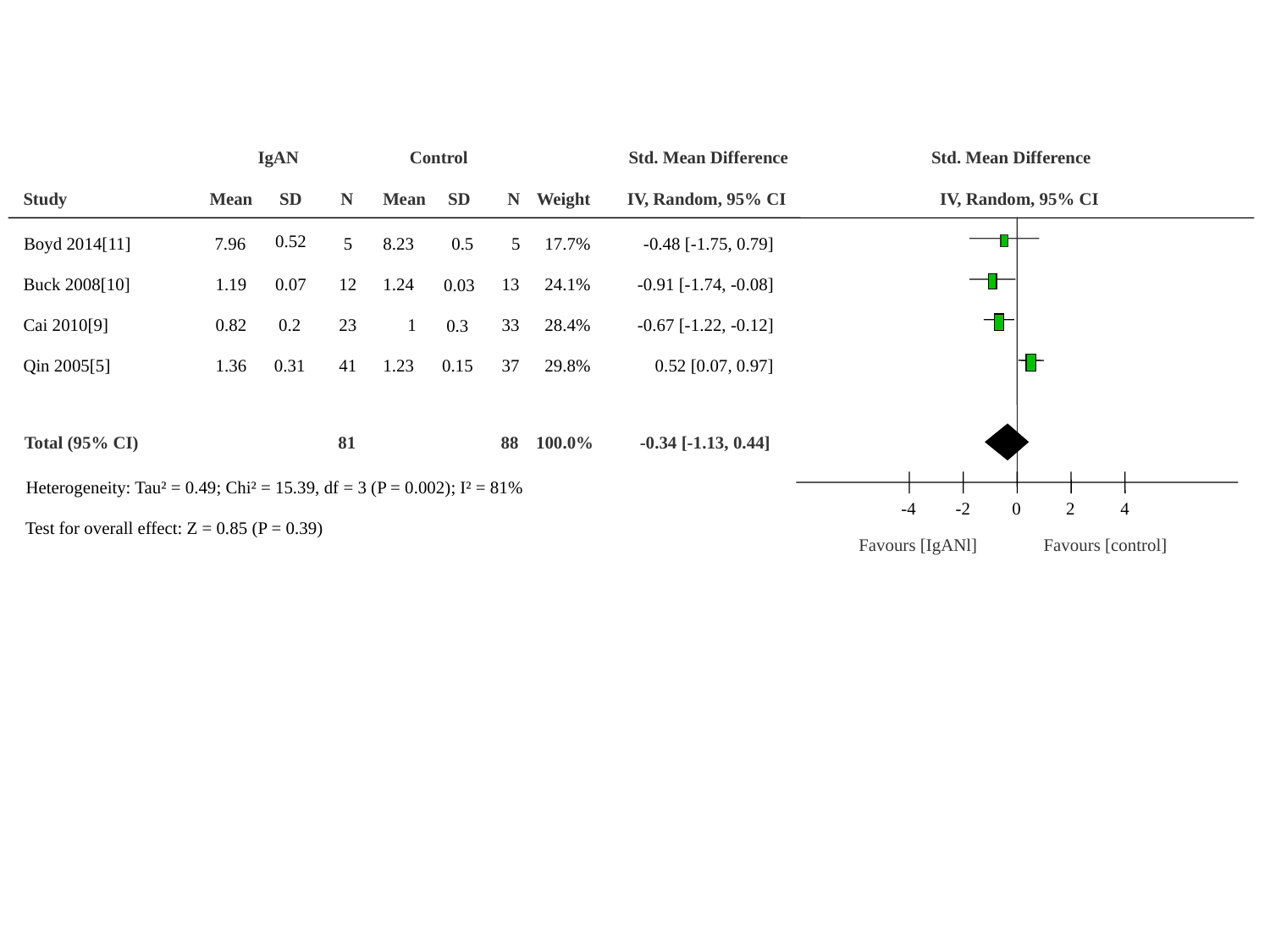

IgAN
Control
Std. Mean Difference
Std. Mean Difference
Study
Mean
SD
N
Mean
SD
N
Weight
IV, Random, 95% CI
IV, Random, 95% CI
0.52
Boyd 2014[11]
7.96
5
8.23
0.5
5
17.7%
-0.48 [-1.75, 0.79]
Buck 2008[10]
1.19
0.07
12
1.24
13
24.1%
-0.91 [-1.74, -0.08]
0.03
Cai 2010[9]
0.2
23
1
33
28.4%
-0.67 [-1.22, -0.12]
0.82
0.3
Qin 2005[5]
1.36
0.31
41
1.23
37
29.8%
0.52 [0.07, 0.97]
0.15
Total (95% CI)
81
88
100.0%
-0.34 [-1.13, 0.44]
Heterogeneity: Tau² = 0.49; Chi² = 15.39, df = 3 (P = 0.002); I² = 81%
-4
-2
0
2
4
Test for overall effect: Z = 0.85 (P = 0.39)
Favours [IgANl]
Favours [control]
