# Supplementary material for: C1GALT1 expression is associated with galactosylation of IgA1 in peripheral B lymphocyte in immunoglobulin a nephropathy
Source: BMC Nephrol. 2020 Jan 15;21:18. doi: 10.1186/s12882-019-1675-5 (PMC6964072; doi:10.1186/s12882-019-1675-5)
Supplement: Supplementary file 4 — Additional file 4: Figure S4. Comparison of the expression of C1GALT1C1 between IgAN and Non-IgAN (A) and Healthy Control(B). (PPTX 88 kb) [file 12882_2019_1675_MOESM4_ESM.pptx]

## Slide 1
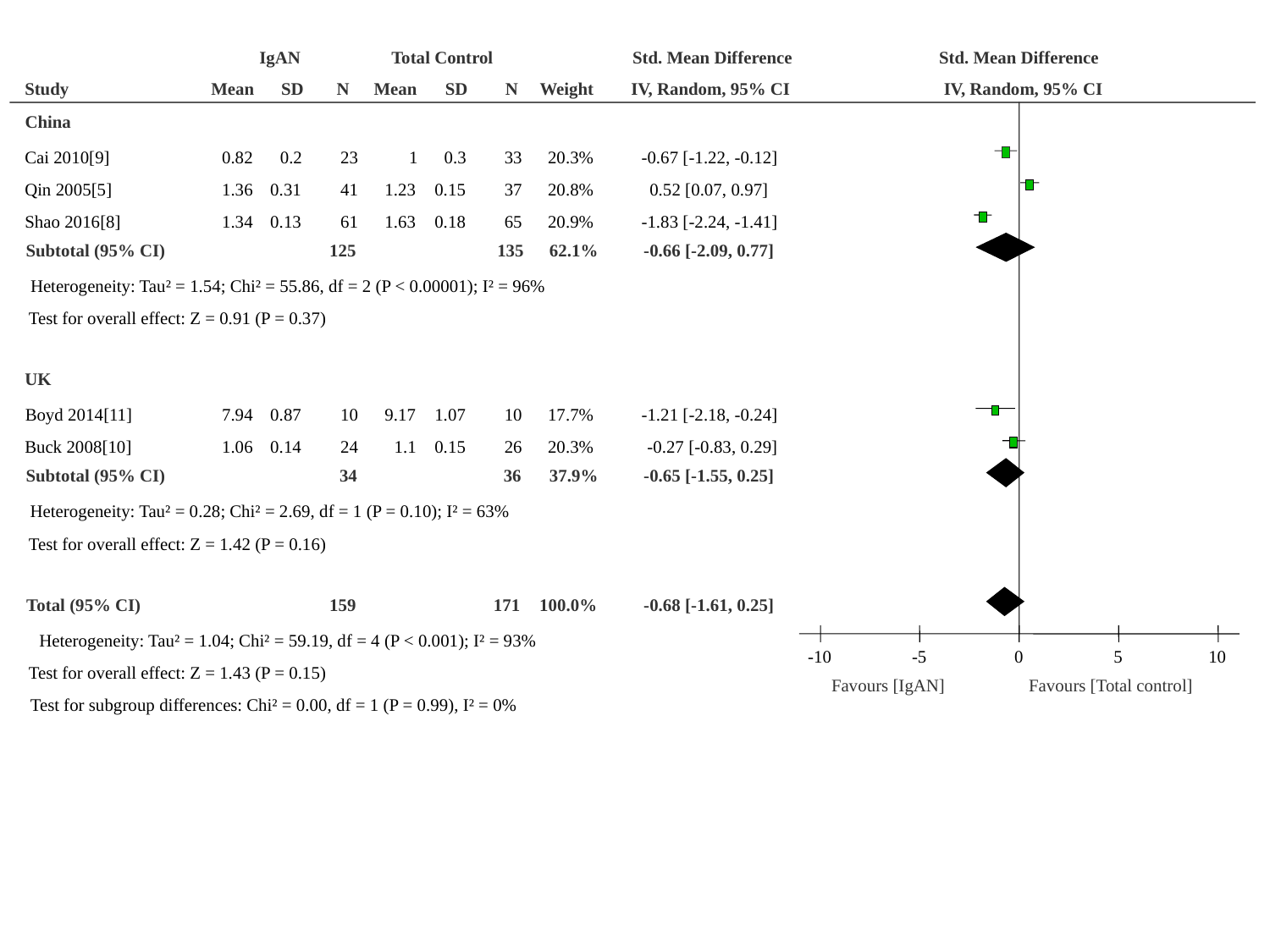

IgAN
Total Control
Std. Mean Difference
Std. Mean Difference
Study
Mean
SD
N
Mean
SD
N
Weight
IV, Random, 95% CI
IV, Random, 95% CI
China
Cai 2010[9]
0.82
0.2
23
1
0.3
33
20.3%
-0.67 [-1.22, -0.12]
Qin 2005[5]
1.36
0.31
41
1.23
0.15
37
20.8%
0.52 [0.07, 0.97]
Shao 2016[8]
1.34
0.13
61
1.63
0.18
65
20.9%
-1.83 [-2.24, -1.41]
Subtotal (95% CI)
125
62.1%
-0.66 [-2.09, 0.77]
135
Heterogeneity: Tau² = 1.54; Chi² = 55.86, df = 2 (P < 0.00001); I² = 96%
Test for overall effect: Z = 0.91 (P = 0.37)
UK
Boyd 2014[11]
7.94
0.87
10
9.17
1.07
10
17.7%
-1.21 [-2.18, -0.24]
Buck 2008[10]
1.06
0.14
24
1.1
0.15
26
20.3%
-0.27 [-0.83, 0.29]
Subtotal (95% CI)
34
36
37.9%
-0.65 [-1.55, 0.25]
Heterogeneity: Tau² = 0.28; Chi² = 2.69, df = 1 (P = 0.10); I² = 63%
Test for overall effect: Z = 1.42 (P = 0.16)
Total (95% CI)
159
171
100.0%
-0.68 [-1.61, 0.25]
Heterogeneity: Tau² = 1.04; Chi² = 59.19, df = 4 (P < 0.001); I² = 93%
-10
-5
0
5
10
Test for overall effect: Z = 1.43 (P = 0.15)
Favours [IgAN]
Favours [Total control]
Test for subgroup differences: Chi² = 0.00, df = 1 (P = 0.99), I² = 0%
